# Racial and Ethnic Disparities in Mpox Cases and Vaccination Among Adult Males — United States, May–December 2022

**DOI:** 10.15585/mmwr.mm7215a4

**Published:** 2023-04-14

**Authors:** Krishna Kiran Kota, Jaeyoung Hong, Carla Zelaya, Aspen P. Riser, Alexia Rodriguez, Daniel L. Weller, Ian H. Spicknall, Jennifer L. Kriss, Florence Lee, Peter Boersma, Elizabeth Hurley, Peter Hicks, Craig Wilkins, Harrell Chesson, Jeniffer Concepción-Acevedo, Sascha Ellington, Ermias Belay, Jonathan Mermin

**Affiliations:** ^1^CDC Mpox Emergency Response Team; ^2^Oak Ridge Institute for Science and Education, Oak Ridge, Tennessee.

As of December 31, 2022, a total of 29,939 monkeypox (mpox) cases* had been reported in the United States, 93.3% of which occurred in adult males. During May 10–December 31, 2022, 723,112 persons in the United States received the first dose in a 2-dose mpox (JYNNEOS)^†^ vaccination series; 89.7% of these doses were administered to males (*1*). The current mpox outbreak has disproportionately affected gay, bisexual, and other men who have sex with men (MSM) and racial and ethnic minority groups (*1*,*2*). To examine racial and ethnic disparities in mpox incidence and vaccination rates, rate ratios (RRs) for incidence and vaccination rates and vaccination-to-case ratios were calculated, and trends in these measures were assessed among males aged ≥18 years (males) (*3*). Incidence in males in all racial and ethnic minority groups except non-Hispanic Asian (Asian) males was higher than that among non-Hispanic White (White) males. At the peak of the outbreak in August 2022, incidences among non-Hispanic Black or African American (Black) and Hispanic or Latino (Hispanic) males were higher than incidence among White males (RR = 6.9 and 4.1, respectively). Overall, vaccination rates were higher among males in racial and ethnic minority groups than among White males. However, the vaccination-to-case ratio was lower among Black (8.8) and Hispanic (16.2) males than among White males (42.5) during the full analytic period, indicating that vaccination rates among Black and Hispanic males were not proportionate to the elevated incidence rates (i.e., these groups had a higher unmet vaccination need). Efforts to increase vaccination among Black and Hispanic males might have resulted in the observed relative increased rates of vaccination; however, these increases were only partially successful in reducing overall incidence disparities. Continued implementation of equity-based vaccination strategies is needed to further increase vaccination rates and reduce the incidence of mpox among all racial and ethnic groups. Recent modeling data (*4*) showing that, based on current vaccination coverage levels, many U.S. jurisdictions are vulnerable to resurgent mpox outbreaks, underscore the need for continued vaccination efforts, particularly among racial and ethnic minority groups.

Data on confirmed and probable cases of mpox were electronically sent by health departments to CDC as part of national case surveillance through a standardized case report form^§^ or to the National Notifiable Diseases Surveillance System.^¶^ Data on JYNNEOS vaccine doses administered were reported to CDC by U.S. jurisdictions (*2*) using a reporting system adapted from protocols originally developed for reporting data on COVID-19 vaccine administration. Case and first-dose vaccination data for males** aged ≥18 years across seven racial and ethnic groups^††^ were included in this analysis; analysis was limited to males because approximately 94% of cases occurred in this group, and 90% of vaccine doses were administered to males. Incidence and vaccination rates were calculated as cases and vaccinations, respectively, per 100,000 MSM because the MSM population was disproportionately affected by the current mpox outbreak. The number of MSM in each racial and ethnic group was estimated by multiplying each racial and ethnic group’s adult male population^§§^ by 3.9%, the estimated percentage of U.S. men who reported having sex with a man in the previous 5 years.^¶¶^ (*5*,*6*).

Incidence and vaccination rates were calculated by race and ethnicity (Asian, non-Hispanic American Indian or Alaska Native [AI/AN], Black, non-Hispanic Native Hawaiian or other Pacific Islander, White, Hispanic, and non-Hispanic multiple races or other races). Because Black, Hispanic, and White males accounted for >88% of mpox cases in males and 83% of JYNNEOS doses administered to males, this report focuses primarily on these three groups. RRs for incidence and vaccination rates were calculated for Black and Hispanic males, with White males as the reference population. The vaccination-to-case ratio was calculated as the number of adult males within a group who received a first dose of vaccine during a given period divided by the number of mpox cases in that group during the same period (*3*).*** This metric was used to ascertain whether higher vaccination rates in racial and ethnic minority groups were proportional to higher mpox incidence in these groups. All disparity measures were calculated for the overall outbreak period (May–December 2022) and for subperiods during 2022: May–June, July, August, September, and October–December (May–June and October–December were combined into single periods, because case and vaccination counts were low during those months). R statistical software (version 4.2.1; R Foundation) was used to conduct all analyses. This activity was reviewed by CDC and was conducted consistent with applicable federal law and policy.^†††^

During May 10–December 31, 2022, a total of 27,946 mpox cases were reported among males aged ≥18 years, including 30.7% in Black males, 29.5% in Hispanic males, and 27.9% in White males (Table 1). Among 648,336 first-dose vaccinations administered to adult males, 11.6%, 20.6% and 51.1% were administered to Black, Hispanic, and White males, respectively. Mpox incidence and vaccination rates were highest in August 2022 for all racial and ethnic groups, with the exception of incidence among White males, which peaked in July. Mpox incidence and vaccination rates subsequently declined through December (Table 2).

**TABLE 1:** Characteristics of adult males* with mpox and those who received JYNNEOS vaccine — United States, May 10–December 31, 2022^†^

Characteristic	No. (%)^§^		
	Mpox cases	JYNNEOS first-dose^¶^ vaccines	JYNNEOS vaccine total doses (first and second doses)
**Total**	**27,946**	**648,336**	**1,039,633**
Age group, yrs**			
18–30	8,598 (30.8)	156,214 (24.1)	235,585 (22.7)
31–40	11,247 (40.2)	200,848 (31.0)	318,614 (30.6)
41–50	5,362 (19.2)	114,283 (17.6)	186,764 (18.0)
51–60	2,299 (8.2)	108,261 (16.7)	181,438 (17.5)
≥61	440 (1.6)	68,730 (10.6)	117,232 (11.3)
**Race and ethnicity^††^**			
AI/AN	103 (0.4)	2,648 (0.4)	4,208 (0.4)
Asian	747 (2.7)	44,259 (6.8)	71,659 (6.9)
Black or African American	8,577 (30.7)	75,496 (11.6)	118,275 (11.4)
NH/OPI	67 (0.2)	1,630 (0.3)	2,580 (0.2)
White	7,793 (27.9)	331,020 (51.1)	545,728 (52.5)
Hispanic or Latino	8,248 (29.5)	133,759 (20.6)	207,910 (20.0)
Multiple races or other	794 (2.8)	38,682 (6.0)	58,699 (5.6)
Missing or unknown	1,617 (5.8)	20,842 (3.2)	30,574 (2.9)
**U.S. Census Bureau region** ^§§^			
Northeast	6,177 (22.1)	159,288 (24.6)	250,150 (24.1)
Midwest	2,761 (9.9)	79,443 (12.3)	129,393 (12.4)
South	11,126 (39.8)	175,657 (27.1)	280,221 (27.0)
West	7,745 (27.7)	230,973 (35.6)	374,697 (36.0)
Puerto Rico	137 (0.5)	2,975 (0.5)	5,172 (0.5)

**Abbreviations:** AI/AN = American Indian or Alaska Native; mpox = monkeypox; NH/OPI = Native Hawaiian or other Pacific Islander.

* Sex and gender data were missing for 442 (1.5%) mpox patients and 12,507 (1.7%) vaccine recipients. Data from these persons were not included.

^†^ Case data updated through January 8, 2023, at 2:00 p.m. Eastern Time. Vaccination data updated through January 10, 2023.

^§^ Percentages might not sum to 100% because of rounding.

^¶^ At least 1 JYNNEOS vaccine dose received.

** Age was missing for 278 (0.9%) mpox patients and 10 (0.001%) vaccine recipients.

^††^ All persons who reported Hispanic or Latino (Hispanic) ethnicity, regardless of race, were categorized as Hispanic. Persons who did not report ethnicity as Hispanic (including missing ethnicity) were categorized as non-Hispanic and self-reported race in the following categories: AI/AN, Asian, Black or African American, NH/OPI, White, and multiple races (more than one race category selected) or other race. Persons with missing data on ethnicity and race were categorized as missing or unknown.

^§§^ https://www2.census.gov/geo/pdfs/maps-data/maps/reference/us_regdiv.pdf. Puerto Rico is not included in any U.S. Census Bureau region.

**TABLE 2:** Mpox incidence and JYNNEOS first-dose administration rates, relative measures of racial and ethnic disparity, and vaccination-to-case ratios among adult males,* by race and ethnicity^†^ and month — United States, May 10–December 31, 2022

Metric	Overall	May–Jun^§^	Jul	Aug	Sep	Oct–Dec
**Mpox case incidence (cases per 100,000 MSM)^¶^**						
AI/AN	**291.2**	2.8	53.7	144.3	62.3	28.3
Asian	**258.5**	15.2	91.4	100.8	31.6	19.8
Black or African American	**1,467.8**	31.5	432.4	608.8	265.4	137.4
NH/OPI	**728.6**	43.5	163.2	348.7	142.2	32.9
White	**252.7**	16.3	89.8	88.4	37.3	21.1
Hispanic or Latino	**907.9**	36.5	286.5	364.9	146.9	76.0
Multiple races or other	**968.6**	28.1	318.5	350.1	168.3	107.1
JYNNEOS vaccine first dose** administration rates (doses administered per 100,000 MSM)^¶^						
AI/AN	**7,486.6**	33.9	939.0	3,283.3	2,019.1	1,416.0
Asian	**15,315.3**	60.9	2,980.5	7,985.0	3,198.3	1,945.3
Black or African American	**12,920.1**	25.5	1,795.3	7,313.1	2,673.9	1,678.4
NH/OPI	**17,725.1**	87.0	3,003.9	9,403.1	3,839.5	2,550.2
White	**10,735.4**	65.2	2,046.0	5,433.1	2,213.5	1,389.8
Hispanic or Latino	**14,723.5**	47.6	2,533.9	7,651.2	3,147.3	2,132.4
Multiple races or other	**47,188.7**	189.1	7,120.7	25,432.9	14,545.1	10,598.3
**Relative disparities in mpox incidence^¶^**						
Black or African American:White RR	**5.8**	1.9	4.8	6.9	7.1	6.5
Hispanic or Latino:White RR	**3.6**	2.2	3.2	4.1	3.9	3.6
Relative disparities in JYNNEOS first-dose** vaccination rates^¶^						
Black or African American:White RR	**1.2**	0.4	0.9	1.3	1.2	1.2
Hispanic or Latino:White RR	**1.4**	0.7	1.2	1.4	1.4	1.5
**Vaccination-to-case ratio^††^**						
AI/AN	**25.7**	12.0	17.5	22.5	31.1	47.0
Asian	**59.2**	4.0	32.6	76.9	90.6	85.2
Black or African American	**8.8**	0.8	4.2	11.8	9.3	11.0
NH/OPI	**24.3**	2.0	18.4	26.2	23.8	66.0
White	**42.5**	4.0	22.8	60.2	55	59.7
Hispanic or Latino	**16.2**	1.3	8.8	20.5	19.4	24.6
Multiple races or other	**48.7**	6.7	22.3	67.6	60.2	59.0
**Overall (aggregated measure)**	**23.8**	**2.7**	**13.2**	**31.0**	**28.2**	**32.8**

**Abbreviations:** AI/AN = American Indian or Alaska Native; mpox = monkeypox; MSM = gay, bisexual, and other men who have sex with men; NH/OPI = Native Hawaiian or other Pacific Islander; RR = rate ratio.

* For incidence and vaccination rates, the numerators were the numbers of cases and first-dose vaccinations, respectively, among all adult males and were not limited to MSM. To obtain meaningful rates, and because MSM were disproportionately affected by the outbreak, the denominators included only MSM, (i.e., incidence and vaccination rates were calculated as cases and vaccinations in all adult males, respectively, per 100,000 MSM). Case data updated through January 8, 2023, at 2:00 p.m. Eastern Time. Vaccination data updated through January 10, 2023.

^†^ All persons who reported Hispanic or Latino (Hispanic) ethnicity, regardless of race, were categorized as Hispanic. Persons who did not report ethnicity as Hispanic (including missing ethnicity) were categorized as non-Hispanic and self-reported race in the following categories: AI/AN, Asian, Black or African American (Black), NH/OPI, White, and multiple races (more than one race category selected) or other race. Persons with missing data on ethnicity and race were categorized as missing or unknown.

^§^ The first mpox case reporting period in this analysis is May 10–June 30. The first date of JYNNEOS vaccination is May 22, 2022. May–June and October–December were combined into single periods because vaccination and case counts were low during those months.

^¶^ Denominators for incidence and vaccine administration rates were MSM aged ≥18 years: CDC Wonder 2021 estimates for males aged ≥18 years were multiplied by 3.9% to calculate MSM by race and ethnicity, resulting in the following: Black = 584,332; Hispanic = 908,470; White = 3,083,431; Asian = 288,985; AI/AN = 35,370; NH/OPI = 9,196; and multiple races or other race = 81,973. The number of cases in each period was subtracted from the denominator for the following period. For vaccination administration rates, the number of adult males who were vaccinated in each period was subtracted from the denominator for the following period.

** At least 1 dose administered; includes those with 1 or 2 doses administered.

^††^ The number of adult males vaccinated within a racial and ethnic group in a period divided by the number of cases in that racial and ethnic group during the same period.

During May 10–December 31, 2022, mpox case incidences were higher among racial and ethnic minority males than those among White males, except Asian males, whose incidence was similar to that among White males. Disparities in mpox incidence peaked in August, particularly among Black (RR = 6.9) and Hispanic (RR = 4.1) males, relative to White males. After incidence peaked in August, disparities decreased slightly through December; however, substantial disparities in incidence remained during October–December.

During May 10–December 31, 2022, overall vaccination rates were typically higher among all racial and ethnic minority adult males, except AI/AN males, than rates among White males. However, early in the outbreak (during May–June), vaccination rates among Black and Hispanic males were lower than those among White males (RR = 0.4 and 0.7, respectively), and by August, rates among Black and Hispanic males exceeded those among White males (RR = 1.3 and RR = 1.4, respectively).

The vaccination-to-case ratio aggregated across all racial and ethnic groups during the full analytic period was 23.8. The highest vaccination-to-case ratios were observed among Asian (59.2) and White (42.5) males, whereas those among Hispanic and Black males were the lowest (16.2 and 8.8, respectively). From the beginning of the outbreak (May–June) to its peak (August), all groups showed increases in vaccination-to-case ratios, reflecting higher vaccination availability during this period. However, across each separate period examined, vaccination-to-case ratios were lowest among Black and Hispanic males (Figure).

**FIGURE:**
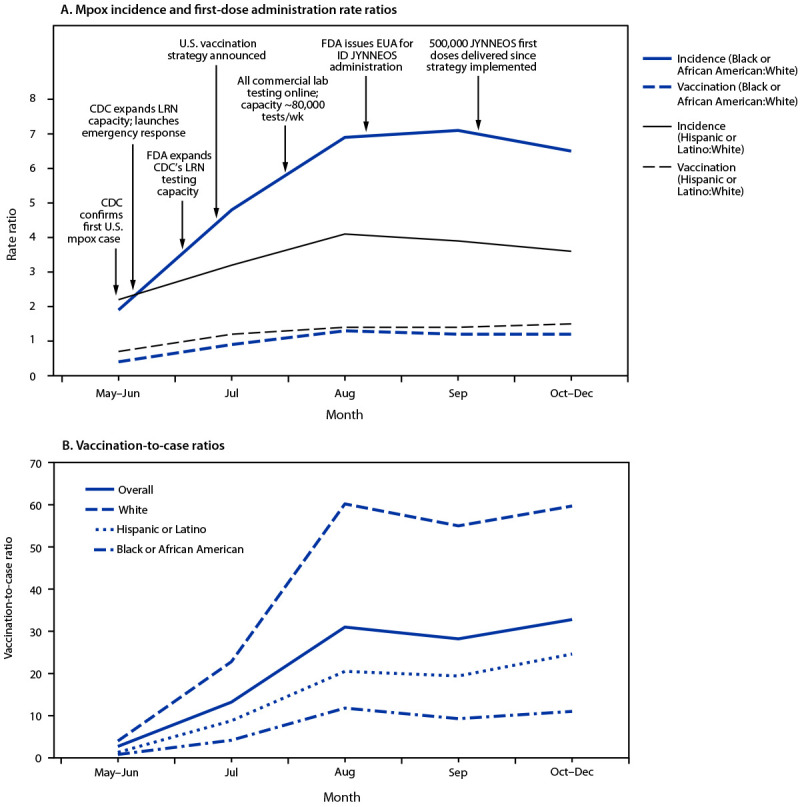
Mpox incidence* and JYNNEOS first-dose administration^†^ rate ratios (A) and vaccination-to-case ratios (B) among adult males, by race and ethnicity^§^ — United States, May 10–December 31, 2022 **Abbreviations**: EUA = Emergency Use Authorization; FDA = Food and Drug Administration; ID = intradermal; LRN = Laboratory Response Network; mpox = monkeypox; MSM = gay, bisexual, and other men who have sex with men. * Cases among males per 100,000 MSM; numerators were the numbers of cases among all adult males and were not limited to MSM. To obtain meaningful rates, and because MSM were disproportionately affected by the outbreak, denominators included only MSM. ^†^ JYNNEOS vaccine doses administered to adult males per 100,000 MSM; numerators were the numbers of doses administered to all adult males and were not limited to MSM. As with incidence, denominators included only MSM. ^§^ All persons who reported Hispanic or Latino (Hispanic) ethnicity, regardless of race, were categorized as Hispanic. Persons categorized as Black or African American or White included only those who did not report Hispanic ethnicity (i.e., those who reported non-Hispanic ethnicity and those with missing ethnicity data).

## Discussion

Analysis of mpox case and vaccine administration data by race and ethnicity provides three important insights. First, there are notable disparities in mpox incidence, characterized by higher rates among males in most racial and ethnic minority groups compared with rates among White males. Second, males in most racial and ethnic minority groups had slightly higher vaccination rates than White males; however, these higher vaccination rates were not sufficiently high to fully offset the disproportionate mpox incidences in these groups. Finally, vaccination-to-case ratios indicated that there is a higher unmet vaccination need among racial and ethnic minority groups, particularly among Black and Hispanic males.

Concerted efforts to increase vaccination among racial and ethnic groups might have contributed to increases in vaccination rates among racial and ethnic minority males (*2*). For example, in September 2022, CDC implemented the mpox vaccine equity pilot program to ensure that vaccination efforts reached communities most affected by the outbreak.^§§§^ In addition, initiation of intradermal vaccine administration on August 9, 2022,^¶¶¶^ increased available doses by three to five–fold, leading to increased access to vaccination (*1*,*7*,*8*).

The vaccination-to-case ratio, a novel measure of vaccination relative to incidence, estimated that approximately 43 White males were vaccinated for each reported mpox case among White males, whereas only nine Black and 16 Hispanic males received mpox vaccination for each reported mpox case within these groups. These findings suggest that the higher vaccination rates among Black and Hispanic males compared with White males (RR = 1.2 and 1.4, respectively) were not commensurate with their higher mpox incidence (RRs = 5.8 and 3.6, respectively). These groups still had higher unmet vaccination needs relative to their mpox incidence. Accordingly, substantially higher vaccination rates among Black and Hispanic males are needed to address the disproportionate incidence and unmet vaccination needs in these groups.

Racial and ethnic disparities in mpox incidence are driven by several factors, including social determinants of health. Black and Hispanic males might face barriers to prevention, including access to information and to mpox vaccines resulting from gaps in dissemination of information, language barriers, racism, homophobia, xenophobia, stigma, discrimination, unemployment, and poverty (*9*). In addition, homophily in sexual partnership selection patterns has been associated with increased disparities in the incidence of sexually transmitted infections such as HIV (*10*) and might be associated with disparities in mpox incidence.

The findings in this report are subject to at least four limitations. First, because of data availability, this analysis was limited to adult males and might not be representative of other population groups. Second, data on the race and ethnicity for 6% of patients and 3% of vaccine recipients were missing, which might limit the interpretation of some racial and ethnic disparities. Third, when calculating reported cases and vaccine recipients, it was not possible to confirm that all males included in the reported case and vaccine recipient numerators were members of the denominator (MSM). Finally, incidence and vaccination rates were calculated using MSM population estimates as the denominator; using population estimates of MSM at increased risk for mpox as the denominator might have substantially increased the estimated incidence and vaccination rates. Using population estimates of the entire adult male population as the denominator would have decreased the estimated incidence and vaccination rates; however, in both scenarios the incidence RRs, vaccination RRs, and vaccination-to-case ratios would be similar to findings in this report.

Findings from this analysis indicate that racial and ethnic disparities in mpox incidence rates peaked during August and September 2022 and decreased slightly toward the end of December 2022, potentially as a result of focused public health efforts. However, racial and ethnic disparities in incidence still exist, and eliminating these disparities remains a priority for continued mpox response efforts. Higher mpox vaccination rates among racial and ethnic minority groups are encouraging, but an unmet vaccination need relative to incidence in racial and ethnic minority groups remains. These findings illustrate the potential impact of and continued need for equity-based vaccination strategies, such as community-specific tailored messaging and expansion of vaccination services to reach racial and ethnic minority groups. Recent modeling data (*4*) showing that, based on current vaccination coverage levels, many U.S. jurisdictions are vulnerable to resurgent mpox outbreaks, underscore the need for continued vaccination efforts, particularly among racial and ethnic minority groups.

**Summary**

**What is already known about this topic?**

Racial and ethnic disparities in monkeypox (mpox) incidence and vaccination have been described.

**What is added by this report?**

During May 10–December 31, 2022, mpox incidence among non-Hispanic Black or African American (Black) and Hispanic or Latino (Hispanic) males was higher than that among non-Hispanic White (White) males. Although overall ≥1-dose JYNNEOS vaccination rates were higher among Black and Hispanic males than White males, they were not high enough to fully offset the disproportionate incidence. Overall, 43 White, nine Black, and 17 Hispanic males were vaccinated for each reported mpox case within these groups.

**What are the implications for public health practice?**

Sustained equity-based strategies, such as tailored messaging and expanding vaccination services to reach racial and ethnic minority groups, are needed to prevent disparities in future mpox outbreaks.

## Footnotes

*
https://www.cdc.gov/poxvirus/monkeypox/response/2022/demographics.html (accessed January 18, 2023).

†
https://www.cdc.gov/poxvirus/monkeypox/response/2022/vaccines_data.html (accessed January 18, 2023).

§
https://www.cdc.gov/poxvirus/monkeypox/health-departments/case-reporting.html

¶
https://www.cdc.gov/nndss/index.html

** For case data, persons who reported their sex at birth as male and gender identity as male were categorized as males. Among persons with information on gender identity but not on sex at birth, those who reported their gender identity as male were categorized as males. Among persons with information on sex at birth and not on gender identity, those who reported their sex at birth as male were categorized as males. Thus, some mpox cases in transgender male persons and gender nonbinary persons might have been included in this analysis. For vaccine administration data, persons who reported their sex at birth as male were categorized as males. Gender identity information was not available for vaccine administration data.

†† All persons who reported Hispanic ethnicity, regardless of race, were categorized as Hispanic. Persons who did not report ethnicity as Hispanic (including missing ethnicity) were categorized as non-Hispanic and self-reported race in the following categories: American Indian or Alaska Native, Asian, Black, Native Hawaiian or other Pacific Islander, White, and multiple races (more than one race category selected) or other race. Persons with missing data on ethnicity and race were categorized as missing or unknown.

§§
https://wonder.cdc.gov/single-race-v2021.html

¶¶ The number of MSM was estimated by assuming 3.9% of adult males in all racial and ethnic groups are MSM, based on a 2012 meta-analysis of four population-based surveys. This value was applied in subsequent studies to estimate the U.S. MSM population. This analysis used the entire MSM population aged ≥18 years as the denominator for mpox case rates and mpox vaccination rates. However, if racial and ethnic minority groups are disproportionately represented in the subpopulation of MSM at highest risk for mpox (e.g., MSM with HIV and MSM with indications for HIV preexposure prophylaxis), the use of subpopulations of MSM with highest risk for mpox as the denominator for mpox incidence rates could mask racial and ethnic incidence disparities. For example, assuming that all MSM at high risk for mpox have the same monthly risk for acquiring mpox, but MSM in racial and ethnic minority groups are more likely to be at high risk for mpox than White MSM, no racial and ethnic incidence disparities would be observed when the analysis was limited to MSM at high risk. Conversely, using the entire MSM population as the denominator for mpox vaccination rates could mask racial and ethnic mpox vaccination disparities among those most in need of vaccination. However, using the same denominator (all MSM) for mpox case rates and mpox vaccination rates allowed for consistency in comparing racial and ethnic disparities in these two outcomes.

*** CIs for disparity measures in reported mpox incidence and vaccination rates were not calculated because the numerators of these rate calculations were the actual number of reported cases and vaccinations, not estimates of the number of cases and vaccinations. Although an estimate (the MSM population size) was used in the denominators of the mpox incidence and vaccination rates, use of the estimated MSM population size affected only the absolute magnitude of the rates, not the relative comparisons of rates across racial and ethnic groups. Because the MSM population was estimated assuming MSM account for 3.9% of adult males in each racial and ethnic group, using the entire adult male population as the denominator would result in incidence and vaccination rates that are approximately 3.9% as high (for all racial and ethnic groups) as the rates when estimated using the MSM population as the denominator and would have practically no effect on the rate ratios and vaccination-to-case ratios.

††† 45 C.F.R. part 46, 21 C.F.R. part 56; 42 U.S.C. Sect. 241(d); 5 U.S.C. Sect. 552a; 44 U.S.C. Sect. 3501 et seq.

§§§
https://www.cdc.gov/poxvirus/monkeypox/health-departments/vaccine-equity-pilot.html

¶¶¶
https://www.cdc.gov/poxvirus/monkeypox/interim-considerations/overview.html
